# Reproductive Women's Knowledge on Possibility of Pregnancy after Birth but before Resumption of Menstruation and Its Associated Factors in Ethiopia: A Population-Based Study Using the 2016 Ethiopian Demographic Health Survey

**DOI:** 10.1155/2022/8520323

**Published:** 2022-08-05

**Authors:** Teshome Gebremeskel Aragie, Girma Seyoum Gedion

**Affiliations:** ^1^Department of Medical Anatomy, Woldia University College of Health Sciences, Woldia, Ethiopia; ^2^Department of Anatomy, Addis Ababa University College of Health Sciences School of Medicine, Addis Ababa, Ethiopia

## Abstract

**Introduction:**

Worldwide, specifically in developing countries, women believe that a woman cannot become pregnant unless she sees her first postpartum menstruation. Due to this knowledge gap, most women did not use any contraceptives till their 1^st^ postpartum menstruation. Hence, about 44% of women were susceptible to unintended pregnancy in the postpartum period. Assessing women knowledge on possibility of pregnancy after giving birth but before returning of menses and its associated factors will help to increase women's recognition on the issue and for early commencement of appropriate postpartum family planning methods to reduce burden of unintended pregnancy.

**Objective:**

To assess the level of knowledge on possibility of pregnancy after giving birth but before returning of menses and its associated factors among reproductive women in Ethiopia.

**Methods:**

A secondary data analysis using the 2016 Ethiopian Demographic Health Survey was employed. Samples were selected using two-stage stratified sampling technique. Descriptive statistics and logistic regressions were used. Adjusted odds ratio (AOR) with 95% confidence interval was used to interpret associations, and a significant association was declared at a *p* value of <0.05.

**Result:**

A total of 15,683 reproductive women aged from 15 to 49 years were included. Of them, about 53% did not know that a woman can get pregnant after giving birth but before resumption of her menstruation. Age being 35 years and above (AOR = 1.50; 95%CI = 1.34, 1.67), educational status of secondary and above (AOR = 1.18; 95%CI = 1.06, 1.32), being ever married (AOR = 1.67; 95%CI = 1.47, 1.89), knowledge of any family planning method (AOR = 1.81; 95%CI = 1.52, 2.16), getting counseling on family planning methods (AOR = 1.41; 95%CI = 1.28, 1.55), and being knowledgeable on their ovulatory cycle (AOR = 1.68; 95%CI = 1.55, 1.82) were found to be significantly associated with being knowledgeable on the issue.

**Conclusion:**

Reproductive women's level of knowledge on the possibility of pregnancy after giving birth but before returning of menses was low. Factors associated with being knowledgeable on the issue were identified. Therefore, strategies should be developed to increase their level of knowledge for reducing unintended pregnancy and its complications by integrating family planning counseling with infant immunization services.

## 1. Introduction

In a woman's life, the period about 1 hour after giving birth to the next 6 weeks is called the “postpartum period or puerperium.” This period is the period of resuming a prepregnancy state of anatomy (structure) and physiology (function) of the reproductive system. Structurally, the uterus will involute and returns to its prepregnancy size; abdominal muscles, vagina, cervix, and other reproductive organs will also tend to be in their prepregnancy status. Physiologically, the cardiovascular system, respiratory system, reproductive system, and other body systems will acquire their prepregnancy functions. Among those, physiology of the reproductive system undergoes a significant change, from arrested to active ovarian cycle [1–3].

It is known that during pregnancy, the ovarian function is suppressed by the high levels of placental steroids. Hence, there will not be ovulation and menstruation during pregnancy [4]. But after delivery or abortion, ovulation will reoccur after some periods of anovulation [5]. The length of this anovulation period, resumption of ovulation, and its relation to the first postpartum menstruation have been the subject of many investigations [6]. Different scholars reported that the first postpartum menstruation may or may not be preceded by ovulation [7–9]. However, most women worldwide, specifically in developing countries, think that postpartum menstruation is always anovulatory, and they believe that a woman cannot become pregnant unless she sees her first postpartum menstruation. Hence, despite their sexual activity, they do not use any contraceptives till their 1^st^ menstruation [10–12]. But the fact is a woman's first ovulation during the postpartum period might occur before her first postpartum menstruation, and it is possible for a woman to get pregnant before the resumption of menstruation. Even up to 44% of women are susceptible to unintended pregnancy in the postpartum period as ovulation can occur before menstruation as early as 28 days postpartum [13].

Unintended pregnancies are always associated with health risks for both the mother and the infant. They might end up with unsafe abortion and maternal mortality [14, 15]. Those pregnancies will also cause a closely spaced interpregnancy interval, which is very dangerous for the infant. When births are closely spaced, there will be overlapping of breastfeeding with pregnancy, which could affect the breastfeeding of the newborn. Consequently, short intervals could indirectly increase the risk of adverse neonatal/infant outcomes through changes in breastfeeding patterns or the composition and/or quantity of breast milk [16]. Evidences indicate that interpregnancy intervals shorter than 18 months are also associated with increased risk of preterm birth, due to increased incidence of cervical incompetence secondary to inadequate time for regaining muscle tone in reproductive tissues [17–19]. Literature had also revealed that pregnancy during the postpartum period is a risk factor for low birth weight, small for gestational age, stillbirth, and increase childhood morbidity and mortality in general. Often while a women encountered gynecological problems, infertility, and reproductive concern, they are at risk to develop psychological diseases such as anxiety and depression and to have a poor quality of life [20, 21]. Additionally, pregnancies during the postpartum period will cause an increased patient burden for health care settings and increased economic burden for the family and the country at large [18, 22–25].

Recognizing the possibility of pregnancy after giving birth but before returning of menses will help mothers to initiate appropriate postpartum family planning methods before involving in sexual activity. For a pregnancy to occur, endocrinological changes of the women need to be synchronized [26]. However, sometimes there might be a catastrophe of conception after giving birth but before returning of women's menses. Even though breastfeeding and family planning specialists do not agree on when lactating women should begin using the progestin-only methods, studies revealed premature initiation of progestin-only pills have paramount importance to prevent accidental pregnancy. Halderman and Nelson revealed that there is no detectable adverse impact on breastfeeding attributable to progestin-only contraceptive methods initiated within the first 3 days of the postpartum period [27]. Other study also recommends that such contraceptive methods should be delayed for at least 3 days after the birth [28]. This is possibly that progesterone withdrawal is the likely stimulus that initiates lactogenesis; it appears necessary for natural progesterone levels to decline to baseline before a progestin-only contraceptive is initiated [28].

This will decrease the burden of unintended pregnancies, which in turn will decrease preterm birth, low birth weight, unsafe abortion, childhood mortality, maternal mortality, and other potential complications of unintended pregnancies [29, 30]. In fact, there is no right or wrong way to feel about getting pregnant after childbirth. But, according to the World Health Organization (WHO) and charity March of Dimes, the safest option is to wait 24 months and at least for 18 months before trying for another baby, respectively [31, 32]. Literature had revealed that in developing countries, if no births occur within 18 months of preceding birth, under five children mortality would drop by 50% [33].

Assessing the reproductive women's knowledge on possibility of pregnancy after giving birth but before returning of menses will help to determine their knowledge level. Identifying factors associated with women's knowledge on possibility of pregnancy after giving birth but before returning of menses will also help policy makers and health care providers to develop factor oriented strategies targeting to increase women's knowledge on this issue, which will help to reduce unintended pregnancies and its complications. Despite this, studies addressing the issue are limited worldwide. As per the investigators knowledge, there is no a study assessing the level of women's knowledge on possibility of pregnancy after giving birth but before returning of menses and its associated factors among reproductive women in Ethiopia. Therefore, this study was aimed to assess the level of knowledge on possibility of pregnancy after giving birth but before returning of menses and its associated factors among reproductive women in Ethiopia using the 2016 Ethiopian Demographic Health Survey.

## 2. Methods

### 2.1. Study Design and Period

A secondary data analysis using the 2016 Ethiopian Demographic and Health Survey (EDHS) (the fourth and most recent DHS in Ethiopia) data, collected from January 18 to June 27, 2016, was employed by applying the principles of cross-sectional study design.

### 2.2. Population

All reproductive women (aged from 15-49 years) living in Ethiopia were the source population, and all reproductive women (aged 15-49 years) living in the selected strata were the study population [34].

### 2.3. Samples and Sampling Procedure

The survey had included a nationally representative sample of reproductive women from the nine regions and two administrative cities in the country selected with a two-stage stratified sampling technique. In the first stage, regions of the country were stratified into urban and rural settings and 645 strata were selected. In the second stage, a fixed number of 28 households per strata area were selected with the probability sampling technique. All reproductive age group women who were usual members of the selected households or who spent the night before the survey in the selected households were eligible for the survey. For this study, all women samples (*n* = 15,683) of the 2016 EDHS were included [34].

### 2.4. Data Collection Tool, Process, and Quality Assurance

Five standardized and validated questionnaires were used for the 2016 EDHS. The questionnaires were adapted from the DHS Program's standard Demographic and Health Survey questionnaires in a way to reflect the population and health issues relevant to Ethiopia. In addition to the use of validated tools in the data collection process, the 2016 EDHS has used well-trained field personnel and followed standardized protocols to ensure data quality [34]. For the purpose of the current study, the women's data from the 2016 EDHS was utilized.

### 2.5. Study Variables

In this study, the variable of interest was that can women get pregnant after birth and before menstrual period among reproductive age group. Independent variables which were considered to be associated to this variable were respondent's age, education, occupation, wealth index, marital status, and knowledge of any family planning, knowledge of ovulatory cycle, family planning counseling, and number of live children were considered depending on their availability in the 2016 EDHS data. Age was categorized into 3 categories after taking the age group of 15–24 in one group as youth based on the United Nations definition of the youth age group. Regarding marital status, according to the 2016 EDHS's definition, women who reported being married or living together with a partner as though married at the time of the survey is considered ever married (“we followed the methods of Kassie et al.”) [35].

### 2.6. Statistical Analysis

Data were analyzed using the SPSS version 24. Descriptive statistics were conducted to describe the summary of the sociodemographic characteristics of the study participants and percentage of knowledgeable and nonknowledgeable women regarding possibility of pregnancy after birth but before resumption of menses. Binary logistic regression analysis was conducted, and variables with a *p* value of less than 0.25 were fitted into the multivariable logistic regression analysis model. Then, a multivariable logistic regression analysis was conducted to examine the association between the dependent and independent variables. Odds ratio with 95% confidence interval was used to interpret the associations, and a *p* value of <0.05 was used to declare significant association.

### 2.7. Operational Definitions

#### 2.7.1. Women Knowledge on Pregnancy after Birth and before the Menstruation Period

Based on Ethiopian Demographic Health Survey data (EDHS, 2016), women knowledge on pregnancy after birth and before the menstruation period was assessed by asking “can women get pregnant after birth and before period?” Women's response was “yes” or “no.” Hence, we took the response “yes” as “knowledgeable women” and “no” for “nonknowledgeable women.”

#### 2.7.2. Ethical Considerations

Before conducting this research, an approval to download and use the EDHS 2016 datasets was obtained from the DHS program. 2016 EDHS was reviewed and approved by the Federal Democratic Republic of Ethiopia Ministry of Science and Technology and the Institutional Review Board of ICF International (“we followed the methods of Kassie et al.”) [35].

#### 2.7.3. Consent

All the participants had given informed written consent about the survey before interviewing, and for adolescents less than 18 years old, consent was obtained from parents/guardians and assented by them. Participation in the survey was completely based on willingness and with full autonomy to participate fully and partially and/or to reject participation at any point of the interview. All participants' information was processed anonymously and is labeled with only identification codes in the EDHS dataset (“we followed the methods of Kassie et al.”) [35].

## 3. Result

The response rate of EDHS 2016 women's survey was 95%. In this DHS analysis, a total of 15,683 reproductive women were included, with the mean women's age of 27.94 ± 9.16 years. Almost greater than 7/10 (72.8%) of the participants were ever married in their life time. Half of the participants (51.3%) have no occupation within the last 12-month period. Nearly 2/3 (65.9%) of the participants were rural residents (Table 1).

### 3.1. Reproductive Women's Knowledge on Possibility of Pregnancy after Birth but before Resumption of Menstruation

**Figure 1:**
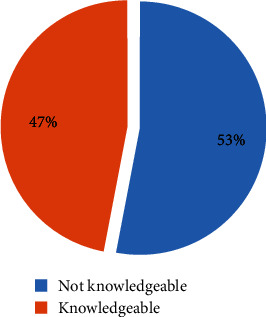
Women's knowledge on possibility of pregnancy after birth but before the resumption of menstruation using Ethiopian Demographic Health Survey 2016 analysis.

Based on the current analysis, from the total of 15,683 women who participated in the survey, greater than half of 8319 (53%) of them were not knowledgeable about the possibility of pregnancy after birth but before the resumption of menstruation. Therefore, less than half of 7364 (47%) participant women were found to be knowledgeable about the possibility of pregnancy after birth but before resumption of menstruation (Figure 1).

### 3.2. Factors Associated with Knowledge of Women on Possibility of Pregnancy after Birth but before Menstruation

On binary logistic regression, eleven [11] variables were entered, and age, marital status, occupation, educational status, knowledge of any family planning method, getting counseling on family planning methods, knowledge of ovulatory cycle, wealth index, and number of live children (parity) were found to be associated with the outcome variable with a *p* value of < 0.25, and those variables were entered into the multivariable logistic regression model for the final decision.

On the final multivariable logistic regression model, age, marital status, occupation, educational status, knowledge of any family planning method, getting counseling on family planning methods, knowledge of ovulatory cycle, wealth index, and number of live children (parity) were found to be significantly associated with knowledge of women on occurrence of pregnancy after birth and before the menstrual period among reproductive age women at a *p* value of <0.05.

In this DHS analysis, the odds of women aged 35 years and above were 1.5 times (AOR = 1.5, 95% CI (1.34, 1.67)) higher as regards knowledge regarding the possibility of pregnancy after giving birth but before the resumption of menstruation than the women of aged 15-24 and 25-34 years. In this analysis, the odds of getting family planning counseling during the last 12 months was 1.81 times (AOR = 1.81, 95% CI (1.52, 2.16)), indicating that these women are more knowledgeable than the women who did not get any family planning counseling during the last 12 months. Women who have been using any family planning methods during their life time had 1.41 times (AOR = 1.41 (1.28, 1.55)) higher knowledge on the possibility of pregnancy after birth but before the resumption of menstrual period. Being ever married women are 1.67 times (AOR = 1.67 (1.47, 1.89)) more knowledgeable than not ever married women about the possibility of pregnancy after birth but before resumption of menstruation (Table 2).

In this DHS analysis, having occupation within the last 12 months is positively associated with knowledge of the women regarding the possibility of pregnancy after birth but before resumption of her period; hence, women having occupation are 1.13 times (AOR = 1.13, 95% (1.05, 1.20)) more knowledgeable than women having no occupation. Rich women were 1.2 times (AOR = 1.21 (1.10, 1.34)) more knowledgeable on the possibility of pregnancy before the 1^st^ menstruation during the postpartum period. In this analysis, educational status is positively associated with women knowledge: hence, women who have secondary school and above educational status were 1.18 times (AOR = 1.18 (1.06, 1.32)) more knowledgeable than women having less than secondary and above educational status. Women having knowledge on their ovulatory cycle were 1.68 times (AOR = 1.68 (1.55, 1.82)) more knowledgeable regarding the possibility of pregnancy after birth but before resumption of menstruation than women having no knowledge on their ovulatory cycle. In the current DHS study, women who have ≥6 live children were 1.45 times (AOR = 1.45, 95% CI (1.23, 1.70)) more knowledgeable on the possibility of pregnancy after child birth but before the resumption of menstruation than those who have no live children during their lifetime (Table 2).

## 4. Discussions

Women's general knowledge of the reproductive system, menstrual cycle, and its associated changes is needed for effective reproductive planning before pregnancy occurs [36]. Different literatures suggested that birth spacing influences the nutritional status of the mother and child. Longer interpregnancy intervals allow for an increase in nutritional reserves of the mother before the following conception. Studies in developing countries revealed that longer birth intervals are associated with lower risk of child malnutrition in some populations [37]. For the mother, a short birth interval may give her insufficient time to recover from the nutritional burden of pregnancy.

In the current DHS analysis, it was revealed that 53% of women are not knowledgeable on the possibility of pregnancy after giving birth but before the resumption of menstruation. This result signifies that greater than half of women are at risk to be pregnant with in their postpartum period. The possible reason might be due to the women's low awareness on their ovulatory period [38], poor knowledge on their fertile window and fertile period [36, 39–42], and difference in breastfeeding practices [43–45]. Women who do not know when they ovulate or how long the egg or sperm could live in a woman's body might have a low insight of their risk for pregnancy, and this could negatively influence their sexual behaviors and contraceptive use [36]. On the other hand, this DHS result was in congruent with the result revealed by Bimrew et al., in which 72.4% of women were not knowledgeable regarding their ovulatory cycle [38].

In this DHS analysis, women who have high income were more knowledgeable on the possibility of pregnancy after giving birth but before resumption of menstruation. The result is parallel with the study conducted by Erickson et al. and Klebanoff, in which women under low socioeconomic status were faced with poor health outcomes due to their poor knowledge of reproductive cycles compared to those with high socioeconomic status [46, 47]. This result is also supported by the studies conducted in USA [48, 49] that there could be economical disparities between women who faced unintended pregnancy; unintended pregnancy for women whose income is below the poverty line is 112 per 1,000 compared with 29 per 1,000 among women whose income is at least twice the poverty line. The possible reason might be due to that women who are under poor economic level are less likely to utilize antenatal care utilization, institutional delivery, and postnatal services [50].

Women who had got family planning counseling were more knowledgeable on the possibility of pregnancy after birth but before resumption of menstruation than their counter parts. The reason might be related to women who got family planning counseling in the last 12 months could have knowledge on their reproductive cycle and fertility period compared to those who did not.

In the current study, women who have ever married were knowledgeable on the possibility of pregnancy after child birth but before resumption of menstruation than not ever married women. This could lead to being unmarried with shorter inter pregnancy intervals yielded increasing risk of infant death [51]. Women who are knowledgeable on their ovulatory cycle were more knowledgeable on the possibility of pregnancy after child birth but before resumption of menstruation. The result is in line with the study conducted in Michigan, USA, revealing that women who understand their menstrual cycle and when they ovulate would be more aware of when they are at risk of pregnancy [36]. The finding is also supported by Laguna et al., in which a women who have an accurate knowledge of their ovulatory cycle is more likely to effectively use the rhythm method of contraception to avoid unintended pregnancies [52].

Even though another factor also affects knowledge of women on the possibility of pregnancy after giving birth but before resumption of menstruation, in this study, women having secondary education and above were more knowledgeable on the possibility of pregnancy after child birth but before resumption of menstruation than uneducated women. This result is confirmed/supported by Simpson, in which lack of or decreased levels of education have been linked to unintended pregnancies and shorter birth intervals [53].

## 5. Conclusions

Reproductive women's level of knowledge on the possibility of pregnancy after giving birth but before returning of menses was low. Factors associated with being knowledgeable on the issue were identified. Therefore, strategies should be developed to increase their level of knowledge for reducing unintended pregnancy and its complications. Moreover, integration of family planning counseling with infant immunization services might be crucial.

## 6. Recommendations and Future Implications

Efforts should be enhanced on contraception counseling prior to discharge and early postpartum visits, which are required to increase early use of effective contraception.

Clinical care practitioners and program managers should apply integrated family planning service counseling and reproductive health education for all women as part of clinical routines, whether during pregnancy, preconception, or postpartum period.

Further providing accurate information on reproductive physiology (ovulation and menstrual cycle) would be beneficial for the women to understand their pregnancy risk, plan their pregnancies, and be aware of their pregnancies early.

Improving knowledge of women and their families regarding the adverse consequences of fertility intervals could be a straightforward intervention for all stakeholders.

## 7. Strength and Limitations of the Study

Quality of the data is assured as the EDHS uses well-trained field personnel, a standardized protocol, and validated tools in the data collection process. However, some of the very important determinants of women knowledge on pregnancy after birth and before menstruation such as postpartum family planning utilization were not included in this study because the relevant pieces of information regarding these variables are not available in the 2016 EDHS data.

## Figures and Tables

**Table 1:** Sociodemographic characteristics of women included under this Ethiopian demographic health analysis 2016.

Variables	Category	Count	Percentage
Age in years	15-24	6401	40.8
	25-34	5089	32.4
	≥35	4196	20.6
Residence	Urban	5348	34.1
	Rural	10335	65.9
Marital status	Not married	4278	27.3
	Ever married	11405	72.8
Educational status	No education	7033	44.8
	Primary education	5213	33.2
	Secondary and above	3437	21.9
Occupation (within the last 12 months)	No occupation	8045	51.3
	Has occupation	7638	48.7
Wealth index	Poor	5940	37.9
	Middle	2002	12.8
	Rich	7741	49.4
Number of live children	0	5539	35.3
	1-5	8053	51.3
	≥6	2091	13.3

**Table 2:** Bivariate and multivariable logistic regression on women's knowledge on the possibility of pregnancy after birth but before resumption of the menstrual period and its associated factors: EDHS 2016 data analysis.

Variables	Level of knowledge (*N* = 15,683)		Odd ratios	
	Not knowledgeable	Knowledgeable		
	8319 (53.0%)	7364 (47.0%)	COR (95% CI)	AOR (95% CI)
Age (in years)				
15-24	4120 (26.3)	2281 (14.5)	1	1
25-34	2420 (15.4)	2666 (17)	1.99 (1.84, 2.14)	1.23 (1.12, 1.35)^∗^
35 years and above	1779 (11.3)	2417 (15.4)	2.45 (2.26, 2.65)	1.50 (1.34, 1.67)^∗^
Educational level				
Not educated	3512 (22.4)	3521 (22.5)	1	1
Primary	3038 (19.4)	2175 (13.9)	0.71 (0.66, 0.76)	0.93 (0.856, 1.02)
Secondary and above	1769 (11.3)	1668 (10.6)	0.94 (0.86, 1.02)	1.18 (1.06, 1.32)^∗^
Marital status				
Not married	2982 (19)	1296 (8.3)	1	1
Ever married	5337 (34)	6068 (38.3)	2.61 (2.42, 2.81)	1.67 (1.47, 1.89)^∗^
Occupation				
No occupation	4498 (28.7)	3547 (22.6)	1	1
Have occupation	3821 (24.4)	3817 (24.3)	1.26 (1.19, 1.34)	1.13 (1.05, 1.20)^∗^
Knowledge of the ovulatory cycle				
Not knowledgeable	6771 (43.2)	5213 (33.2)	1	1
Knowledgeable	1548 (9.9)	2151 (13.7)	1.80 (1.67, 1.94)	1.68 (1.55, 1.82)^∗^
Wealth index				
Poor	3309 (21.1)	2631 (16.8)	1	
Middle	1069 (6.8)	933 (5.9)	1.09 (0.99, 1.21)	1.07 (0.962, 1.19)
Rich	3941 (25.1)	3800 (24.2)	1.21 (1.13, 1.29)	1.21 (1.10, 1.34)^∗^
Knowledge of any family planning				
Not knowledgeable	515 (3.3)	194 (1.2)	1	1
Knowledgeable	7804 (49.8)	7170 (45.7)	2.43 (2.06, 2.88)	1.81 (1.52, 2.16)^∗^
Number of live children (parity)				
0	3704 (23.6)	1835 (11.7)	1	1
1-5	3684 (23.5)	4369 (27.9)	2.39 (2.23, 2.57)	1.43 (1.26, 1.61)^∗^
≥6	931 (5.9)	1160 (7.4)	2.51 (2.26, 2.78)	1.45 (1.23, 1.70)^∗^
Getting family planning counseling				
No	7424 (47.3%)	5976 (38.1%)	1	1
Yes	895 (5.7%)	1388 (8.85%)	1.97 (1.76,210)	1.41 (1.28, 1.55)^∗^

## Data Availability

The data is EDHS (Ethiopian Demographic Health Survey Data). We got it from the owners with formal request. If anybody needs the data, we can provide it after we get permission from the owners.

## Abbreviations

AOR: Adjusted odds ratio
OR: Odds ratio
DHS: Demographic Health Survey
EDHS: Ethiopian Demographic Health survey
WHO: World Health Organization.

## References

[1] Chauhan G., Tadi P. (2020). *Physiology, postpartum changes*.

[2] Dawidowicz A., Krajewska K., Krajewska-Kułak E. (2004). Women’s knowledge of health behaviors in the puerperium. *Wiadomosci Lekarskie (Warsaw, Poland: 1960)*.

[3] Panda S., Das A., Mallik A., Baruah S. R. (2021). *Normal Puerperium*.

[4] Mikhail G., Allen W. (1967). Ovarian function in human pregnancy. *American Journal of Obstetrics and Gynecology*.

[5] Jackson E., Glasier A. (2011). Return of ovulation and menses in postpartum nonlactating women: a systematic review. *Obstetrics and Gynecology*.

[6] Said S., Johansson E. D., Gemzell C. (1974). Return of ovulation during the postpartum period. *Acta Obstetricia et Gynecologica Scandinavica*.

[7] Davis E. (1946). The clinical use of oral basal temperatures. *Journal of the American Medical Association*.

[8] Grünberger V. (1948). The first bleeding after birth and miscarriage. *Wiener Klinische Wochenschrift*.

[9] Malkani P., Mirchandani J. (1960). "Ovulation during lactation" as determined by endometrial biopsy. *Journal of Obstetrics and Gynaecology of India*.

[10] Borda M., Winfrey W. (2010). *Postpartum Fertility and Contraception: An Analysis of Findings from 17 Countries*.

[11] Kaydor V. K., Adeoye I. A., Olowolafe T. A., Adekunle A. O. (2018). Barriers to acceptance of post-partum family planning among women in Montserrado County, Liberia. *Liberia. Nigerian Postgraduate Medical Journal.*.

[12] Tran N. T., Yameogo W. M. E., Gaffield M. E. (2018). Postpartum family-planning barriers and catalysts in Burkina Faso and the Democratic Republic of Congo: a multiperspective study. *Open Access Journal of Contraception*.

[13] Mwalwanda C. S., Black K. I. (2013). Immediate post-partum initiation of intrauterine contraception and implants: a review of the safety and guidelines for use. *Australian and New Zealand Journal of Obstetrics and Gynaecology*.

[14] Yazdkhasti M., Pourreza A., Pirak A., Abdi F. (2015). Unintended pregnancy and its adverse social and economic consequences on health system: a narrative review article. *Iranian Journal of Public Health*.

[15] Dehingia N., Dixit A., Atmavilas Y. (2020). Unintended pregnancy and maternal health complications: cross-sectional analysis of data from rural Uttar Pradesh, India. *BMC Pregnancy and Childbirth*.

[16] Conde-Agudelo A., Rosas-Bermudez A., Castaño F., Norton M. H. (2012). Effects of birth spacing on maternal, perinatal, infant, and child health: a systematic review of causal mechanisms. *Studies in Family Planning*.

[17] Conde-Agudelo A., Rosas-Bermúdez A., Kafury-Goeta A. C. (2006). Birth spacing and risk of adverse perinatal outcomes. *Journal of the American Medical Association*.

[18] Wendt A., Gibbs C. M., Peters S., Hogue C. J. (2012). Impact of increasing inter-pregnancy interval on maternal and infant health. *Paediatric and Perinatal Epidemiology*.

[19] Chen S.-F., Shau W.-Y., Hsieh C.-C., Hsu J.-J., Hung T.-H. (2005). The impact of interpregnancy interval and previous preterm birth on the subsequent risk of preterm birth. *The Journal of the Society for Gynecologic Investigation: JSGI.*.

[20] La Rosa V., Valenti G., Sapia F., Gullo G., Rapisarda A. (2018). Psychological impact of gynecological diseases: the importance of a multidisciplinary approach. *Ital J Gynaecol Obstet.*.

[21] Gullo G., Cucinella G., Perino A. (2021). The gender gap in the diagnostic-therapeutic journey of the infertile couple. *International Journal of Environmental Research and Public Health*.

[22] Omani-Samani R., Ranjbaran M., Mohammadi M. (2019). Impact of unintended pregnancy on maternal and neonatal outcomes. *The Journal of Obstetrics and Gynecology of India.*.

[23] Gharaee M., Baradaran H. R. (2020). Consequences of unintended pregnancy on mother and fetus and newborn in north-east of Iran. *The Journal of Maternal-Fetal & Neonatal Medicine.*.

[24] Bener A., Saleh N. M., Salameh K. M. K. (2012). The impact of the interpregnancy interval on birth weight and other pregnancy outcomes. *Revista Brasileira de Saúde Materno Infantil.*.

[25] Gipson J. D., Koenig M. A., Hindin M. J. (2008). The effects of unintended pregnancy on infant, child, and parental health: a review of the literature. *Studies in Family Planning*.

[26] Riva A., Buzzaccarini G., Vitagliano A., Laganà A. S., Cucinella G., Gullo G. (2022). Progesterone: the key to success?. *Clinical and Experimental Obstetrics & Gynecology*.

[27] Halderman L. D., Nelson A. L. (2002). Impact of early postpartum administration of progestin-only hormonal contraceptives compared with nonhormonal contraceptives on short-term breast-feeding patterns. *American Journal of Obstetrics and Gynecology*.

[28] Kennedy K. I., Short R. V., Tully M. R. (1997). Premature introduction of progestin-only contraceptive methods during lactation. *Contraception*.

[29] Sridhar A., Salcedo J. (2017). Optimizing maternal and neonatal outcomes with postpartum contraception: impact on breastfeeding and birth spacing. *Maternal health, neonatology and perinatology.*.

[30] Cameron S. (2014). Postabortal and postpartum contraception. *Best Practice & Research. Clinical Obstetrics & Gynaecology*.

[31] Organization WH (2007). *Report of a WHO technical consultation on birth spacing: Geneva, Switzerland 13-15 June 2005*.

[32] Johnson K. (2014). *March of Dimes Calls for 50% Reduction in preterm births*.

[33] Fotso J. C., Cleland J., Mberu B., Mutua M., Elungata P. (2013). Birth spacing and child mortality: an analysis of prospective data from the Nairobi urban health and demographic surveillance system. *Journal of Biosocial Science*.

[34] Central Statistical Agency (CSA) [Ethiopia] and ICF (2016). *Addis Ababa E, and Rockville*.

[35] Mengesha Kassie A., Beletew Abate B., Wudu Kassaw M., Gebremeskel A. T. (2020). Prevalence of underweight and its associated factors among reproductive age group women in Ethiopia: analysis of the 2016 Ethiopian Demographic and Health Survey data. *Journal of Environmental and Public Health*.

[36] Ayoola A. B., Zandee G. L., Adams Y. J. (2016). Women's knowledge of ovulation, the menstrual cycle, and its associated reproductive changes. *Birth*.

[37] Dewey K. G., Cohen R. J. (2007). Does birth spacing affect maternal or child nutritional status? A systematic literature review. *Maternal & Child Nutrition*.

[38] Getahun M. B., Nigatu A. G. (2020). Knowledge of the ovulatory period and associated factors among reproductive women in Ethiopia: a population-based study using the 2016 Ethiopian Demographic Health Survey. *International Journal of Women's Health*.

[39] Mahey R., Gupta M., Kandpal S. (2018). Fertility awareness and knowledge among Indian women attending an infertility clinic: a cross-sectional study. *BMC Women's Health*.

[40] Hampton K., Mazza D. (2015). Fertility-awareness knowledge, attitudes and practices of women attending general practice. *Australian Family Physician*.

[41] Pedro J., Brandão T., Schmidt L., Costa M. E., Martins M. V. (2018). What do people know about fertility? A systematic review on fertility awareness and its associated factors. *Upsala Journal of Medical Sciences*.

[42] Beeman P. (2010). Natural family planning in education and practice. *The Linacre Quarterly.*.

[43] Howie P. W., McNEILLY A. S., Houston M., Cook A., Boyle H. (1982). Fertility after childbirth: post-partum ovulation and menstruation in bottle and breast feeding mothers. *Clinical Endocrinology*.

[44] Howie P. W., McNeilly A. S. (1982). Effect of breast-feeding patterns on human birth intervals. *Reproduction*.

[45] Howie P. W., McNEILLY A. S., Houston M., Cook A., Boyle H. (1982). Fertility after childbirth: infant feeding patterns, basal PRL levels and post-partum ovulation. *Clinical Endocrinology*.

[46] Klebanoff M. A. (1999). The interval between pregnancies and the outcome of subsequent births. *Mass Medical Soc*.

[47] Erickson J., Bjerkedal T. (1979). Interval between pregnancies. *The Lancet.*.

[48] Dehlendorf C., Rodriguez M. I., Levy K., Borrero S., Steinauer J. (2010). Disparities in family planning. *American Journal of Obstetrics and Gynecology*.

[49] Finer L. B., Henshaw S. K. (2006). Disparities in rates of unintended pregnancy in the United States, 1994 and 2001. *Perspectives on Sexual and Reproductive Health*.

[50] Byrd J. F. (2010). *The Integration of Medical and Public Health Education: Historical Perspectives, Review of Current MD-MPH Programs, and Research Plan for the Evaluation of the Health Care and Prevention Program*.

[51] Auger N., Daniel M., Platt R. W., Luo Z.-C., Wu Y., Choinière R. (2008). The joint influence of marital status, interpregnancy interval, and neighborhood on small for gestational age birth: a retrospective cohort study. *BMC Pregnancy and Childbirth*.

[52] Laguna E. P., Po A. L. C., Perez A. E., Kanter A. (2000). *Contraceptive Use Dynamics in the Philippines: Determinants of Contraceptive Method Choice and Discontinuation: Population Institute*.

[53] Lee A. (2009). *The Human and Societal Costs of Short Term Birth Intervals*.

